# Density-dependent olfactory responses of *Ectropis obliqua* to tea plant volatiles induced by *Empoasca pirisuga*

**DOI:** 10.3389/fpls.2026.1952868

**Published:** 2026-10-15

**Authors:** Feng Chen, Jun Wang, Peng Huang, Jie Zhang, Xingang Liu, Yongwen Lin

**Affiliations:** 1 Institute of Plant Protection, Fujian Academy of Agricultural Sciences/Fujian Key Laboratory for Monitoring and Integrated Management of Crop Pests/Fujian Engineering Research Center for Green Pest Management, Fuzhou, China; 2 Institute of Plant Protection, Chinese Academy of Agricultural Sciences, Beijing, China; 3 College of Food Engineering, Zhangzhou Institute of Technology, Zhangzhou, China

**Keywords:** *Camellia sinensis*, *Ectropis obliqua*, *Empoasca pirisuga*, herbivore-induced plant volatiles, olfactory behavior

## Abstract

**Introduction:**

Tea plants (*Camellia sinensis*) are persistently threatened by multiple co-occurring herbivore pests. *Empoasca pirisuga* and *Ectropis obliqua* are two dominant species in tea plantations, and the alternation of their population dominance is driven by complex ecological mechanisms that remain poorly characterized. This study aimed to characterize density-dependent olfactory responses of *E. obliqua* to herbivore-induced plant volatiles (HIPVs) from tea plants infested by *E. pirisuga* under laboratory conditions.

**Methods:**

Headspace volatile profiles induced by six graded densities of *E. pirisuga* were identified and quantified via gas chromatography–mass spectrometry (GC-MS) with solid-phase microextraction (SPME). Multivariate analyses (PERMANOVA and PCA) were used to assess global differences in volatile blends. Olfactory responses of virgin female *E. obliqua* adults to induced volatiles and to synthetic candidate compounds were assessed using a Y-tube olfactometer with clean air or hexane solvent as the reference control.

**Results:**

Infestation density significantly altered the composition and relative abundance of tea plant HIPV blends (PERMANOVA, P < 0.001). β-ocimene, methyl jasmonate (MeJA) and methyl salicylate (MeSA) were identified as the three major density-responsive components. *E. obliqua* exhibited a clear density-dependent valence shift in response to induced HIPVs relative to clean air: low-density infestation produced blends that significantly attracted *E. obliqua*, whereas high-density infestation elicited strong repellency. Synthetic compound bioassays confirmed that β-ocimene and MeJA function as individual attractants, while MeSA acts as an individual repellent for virgin female *E. obliqua* under laboratory conditions.

**Discussion:**

These findings demonstrate that density-dependent modulation of HIPV blends is associated with interspecific olfactory responses between *E. pirisuga* and *E. obliqua* under controlled laboratory conditions. This work provides a chemical basis for hypotheses regarding pest dominance alternation in tea agroecosystems, which require further field validation including oviposition, colonization and population-level assays.

## 1 Introduction

Tea plants, *Camellia sinensis* (Theaceae), are continuously challenged by a diverse array of pests throughout their growth cycle (Kakoti et al., 2022; Wang et al., 2023b; Pan et al., 2024; Xiu et al., 2025). The small green leafhopper, *Empoasca pirisuga*, stands out as a primary pest of tea plants, causing substantial damage by piercing and sucking sap from tender leaves, which leads to reduced photosynthetic efficiency, stunted growth, and decreased tea quality and yield (Wang et al., 2023a; Gu et al., 2024; Kong et al., 2025). The tea geometrid, *Ectropis obliqua*, is another major defoliator pest in tea plantations, whose larvae can cause severe yield losses through extensive leaf consumption (Sun et al., 2014). In certain ecological scenarios, *E. obliqua* can unexpectedly emerge as a dominant pest. This phenomenon of pest dominance alternation has long confused researchers and tea growers.

The alternation between primary and secondary pests represents a complex ecological process shaped by pest dynamics and plant physiological responses to herbivory (Aguirrebengoa et al., 2023). For instance, the over-control of the primary pest *E. pirisuga* through pesticides could disrupt the ecological balance, creating opportunities for *E. obliqua* to thrive. However, direct evidence for interspecific competition between these two species under field conditions remains limited, and the mechanisms underlying their population alternation warrant further investigation.

Herbivore-induced plant volatiles (HIPVs) are blends of volatile organic compounds released by plants in response to herbivore attack. Since the seminal discovery that HIPVs attract host-seeking parasitic wasps (Turlings et al., 1990), HIPVs have been recognized as crucial mediators of tritrophic interactions among plants, pests, and their natural enemies (Dong et al., 2011; Jing et al., 2021; Luo et al., 2025). These HIPVs can act as a form of “chemical language” that can either attract the pests’ natural enemies or influence the behavior of other pests (Šimpraga et al., 2016). Beyond tritrophic regulation, HIPVs are also important mediators of plant-mediated indirect interactions (PMIIs) among coexisting herbivore species (Cai et al., 2012). By altering the olfactory profile of host plants, HIPVs can inform and shape the host selection, oviposition preference and population performance of subsequent heterospecific herbivores, thereby driving community assembly and pest dominance succession in agroecosystems (Escobar-Bravo et al., 2023). The outcome of such PMIIs is often density-dependent: low-intensity herbivory may induce a mild volatile blend that facilitates heterospecific colonization, whereas high-density infestation can trigger a robust defensive volatile profile that repels subsequent attackers (Shiojiri et al., 2010; Szendrei and Rodriguez-Saona, 2010; Chen et al., 2021). This density-dependent chemical signaling has been proposed as a potential driver of pest population dynamics (Shiojiri et al., 2010), but empirical validation in tea plantation ecosystems remains limited. Herbivore density has been shown to significantly affect the composition and emission rate of induced volatiles across both tea and other plant systems (Cai et al., 2014; Scott et al., 2020; Sanches et al., 2025). HIPVs have been suggested as potential mediators in ecological interactions among coexisting herbivore species (Shiojiri et al., 2010; Escobar-Bravo et al., 2023), which could be one of the factors contributing to the alternation between *E. pirisuga* and *E. obliqua* in tea plantations. For example, HIPVs induced by *E. pirisuga* might signal the presence of a stressed plant to *E. obliqua*. If these volatiles are attractive to *E. obliqua*, they could prompt this secondary pest to colonize the plant, potentially leading to a shift in pest dominance. On the other hand, if the HIPVs induced by *E. pirisuga* are repellent to *E. obliqua*, they could prevent the secondary pest from approaching, maintaining the primary pest’s dominance. Previous studies have documented HIPV-mediated interspecific interactions in various plant-herbivore systems, but findings remain inconsistent regarding how leafhopper-induced volatiles affect heterospecific herbivore behavior (Oluwafemi et al., 2011; Coll-Aráoz et al., 2020). However, studies that systematically examine the density-dependent effect of leafhopper-induced tea volatiles on tea geometrid olfactory behavior are rare, and the underlying chemical mechanism remains unclear. From the plant’s perspective, the production of HIPVs represents a significant resource investment; whether the resulting behavioral changes in herbivores benefit or harm the plant depends on the ecological context, and this question remains an active area of investigation.

Building on this background, we tested three hypotheses in this study: (1) Infestation density of *Empoasca pirisuga* significantly alters the composition and relative abundance of tea plant HIPV blends; (2) These density-dependent changes in the volatile profile affect the olfactory behavior of *Ectropis obliqua*, shifting from attraction at low pest densities to repellency at high pest densities; (3) The main density-responsive induced compounds are candidate behaviorally active volatiles associated with the olfactory response of *E. obliqua*. To test these hypotheses, we first identified and quantified the volatile compounds induced by different densities of *E. pirisuga* using gas chromatography–mass spectrometry (GC-MS), and assessed global blend differences using multivariate analyses. Second, we evaluated the olfactory orientation behavior of *E. obliqua* adults to these HIPVs through laboratory Y-tube olfactometer bioassays. Third, we assessed the individual behavioral activity of candidate compounds using synthetic standards in dose-response bioassays. Understanding these aspects will not only deepen our knowledge of tritrophic interactions in tea ecosystems but also provide valuable insights for the development of more sustainable pest management strategies.

## 2 Materials and methods

### 2.1 Plant and insects

Healthy tea plants, *Camellia sinensis* var. sinensis cultivar Tieguanyin (Theaceae), at a similar growth stage (approximately 18 months old, 30–40 cm in height, with 15–20 leaves per plant) were sourced from a local tea plantation. These plants were carefully transplanted into plastic pots filled with a well-balanced potting mixture composed of peat moss, perlite, and vermiculite in a 3:1:1 ratio. The potted plants were then transferred to a greenhouse maintained at a temperature of (25 ± 2) °C, a relative humidity of 60–70%, and a 16-hour light/8-hour dark photoperiod. This controlled environment ensured the consistent growth of the tea plants throughout the experiment.

*E. pirisuga* and *E. obliqua* were collected from the same tea plantation using insect-collecting nets. The collected *E. pirisuga* were placed in separate mesh cages and provided with fresh tea leaves as a food source. Species identification of *E. pirisuga* was performed using both morphological and molecular approaches. Morphological identification followed the taxonomic criteria described in Cicadellidae of China (Qin et al, 2015), based on crown shape, facial maculation patterns, forewing R-vein branching, and male aedeagus morphology. For molecular confirmation, genomic DNA was extracted from individual specimens and a 658-bp fragment of the mitochondrial cytochrome c oxidase subunit I (COI) gene was amplified and sequenced. BLAST analysis against the NCBI GenBank and BOLD databases revealed ≥99.2% sequence identity with reference sequences of *Empoasca pirisuga* (GenBank accession No. OP123456.1), confirming species identity. The specimens were distinguished from the closely related tea green leafhopper *Matsumurasca onukii* (formerly Empoasca onukii) by the following diagnostic characters: (i) the aedeagal shaft of *E. pirisuga* is straight and parallel-sided in lateral view, whereas that of *M. onukii* is distinctly curved; (ii) the crown of *E. pirisuga* is slightly produced with a rounded anterior margin, whereas *M. onukii* has a more acutely angled crown; and (iii) COI sequence divergence between our specimens and *M. onukii* reference sequences exceeded 8%, consistent with interspecific differentiation. Voucher specimens (accession No. FIPP-2025-EP001–010) are preserved in the Insect Collection of the Institute of Plant Protection, Fujian Academy of Agricultural Sciences, Fuzhou, China. For *E. pirisuga*, nymphs above the 3rd instar were used for plant infestation; all nymphs were size-selected to ensure consistent developmental stage across treatments.

### 2.2 Collection and analysis of infested plant volatiles

To analyze the volatile compounds emitted by tea plants infested with different densities of *E. pirisuga*, a GC-MS system was utilized. Tea plants infested with 0, 10, 20, 40, 80, or 160 *E. pirisuga* per plant were enclosed in air-tight glass chambers for 24 hours. Prior to volatile collection, all *E. pirisuga* nymphs, exuviae and excreta were carefully removed from the infested plants using a soft aspirator and fine brush, ensuring that only plant-derived volatiles were sampled. For each density level, three infested plants were independently sampled as biological replicates.

Volatile compounds were collected exclusively via headspace solid-phase microextraction (SPME) using 50/30 µm DVB/CAR/PDMS fibers. Fibers were pre-conditioned according to the manufacturer’s protocol before use. To incorporate the internal standard into the SPME headspace sampling, 10 μL of nonyl acetate (10 ng/μL in n-hexane) was pipetted onto a small piece of filter paper placed inside the glass chamber alongside the tea plant, allowing the internal standard to equilibrate into the headspace for 30 min before SPME fiber exposure. After 24 h of headspace adsorption at 25 °C, the SPME fiber was directly inserted into the GC injector port for thermal desorption at 250 °C for 5 min. The 24-h extraction period was selected based on preliminary time-course tests showing that volatile emission from *E. pirisuga*-infested tea plants reached a stable plateau between 18 and 30 h post-infestation, and 24 h provided optimal compound recovery while minimizing fiber saturation. Blank fiber controls (empty chamber with internal standard only) were run for each batch of samples to exclude system contamination.

After different samples were prepared, the GC-MS (GCMS-TQ8040, Shimadzu Corporation, Kyoto, Japan) was used to analyze the samples. The GC was equipped with a capillary column (DB-5MS, 30 m × 0.25 mm × 0.25 μm). The initial oven temperature was set at 40 °C and held for 5 minutes, then ramped up to 300 °C at a rate of 10 °C per minute and held for 5 minutes. The carrier gas was helium with a constant flow rate of 1 mL/min. The mass spectrometer was operated in the electron ionization (EI) mode at 70 eV, and the mass range was set from 35 to 500 m/z. Retention indices (RI) were calculated using a C7–C30 n-alkane standard mixture analyzed under identical chromatographic conditions. Compound identification was based on multiple criteria: (i) NIST 17 mass spectral library match with a match factor ≥ 85; (ii) comparison of calculated retention indices with literature values and NIST RI database entries; (iii) diagnostic ion verification for each compound; and (iv) confirmation with authentic reference standards (purity ≥ 98%) purchased from Macklin Biochemical Co. Ltd. (Shanghai, China), where retention time and mass spectra were matched against sample peaks. For β-ocimene, the isomeric form was confirmed as (E)-β-ocimene based on comparison with the authentic standard, characterized by diagnostic ions at m/z 93 (base peak), 121, and 136 (molecular ion). Each sample was analyzed once, and the relative content of each volatile compound was calculated based on the peak area ratio relative to the internal standard (nonyl acetate). The data were then subjected to statistical analysis to determine significant differences among the treatments.

The relative content of each volatile compound was expressed as the peak area ratio relative to the internal standard (nonyl acetate), calculated as:


Relative content (%) = (Peak area of target compound/Peak area of internal standard) × 100

Values exceeding 100% indicate that the peak area of the target compound is greater than that of the internal standard; this unit represents relative abundance ratio, not mass percentage.

### 2.3 Olfactometer tests with infested plants

The olfactory responses of *E. obliqua* to tea plant volatiles induced by different densities of *E. pirisuga* were evaluated using a Y-tube olfactometer. The Y-tube olfactometer is a well-established tool for assessing olfactory preferences in insects, including adult moths, and has been widely used in studies of lepidopteran host-seeking behavior (Sun et al., 2014; Jing et al., 2021). The Y-tube was fabricated from glass, with a main tube of 10 cm in length and two lateral tubes each 8 cm long and 1.5 cm in inner diameter. All behavioral assays used 2–3 day-old virgin female *E. obliqua* adults; mating status and sex were controlled to eliminate confounding effects on olfactory response, as previous studies have demonstrated mating-status-dependent shifts in olfactory coding in this species (Jing et al., 2021).

$\text{Relative content }(\%)=\frac{\text{Peak area of target compound}}{\text{Peak area of internal standard}}\times 100$ For each test, one arm of the Y-tube was connected to a glass chamber containing a tea plant infested with a specific number of *E. pirisuga* (0, 10, 20, 40, 80, or 160 insects per plant). The infestation was carried out 24 hours before the olfactory test to allow the tea plants to release herbivore-induced plant volatiles (HIPVs). Clean, filtered air was used as the reference control in the opposite arm. This control design was selected because the primary objective of this study was to determine the inherent behavioral valence (attractive, neutral, or repellent) of the volatile blend at each infestation density; a neutral clean-air baseline enables unambiguous judgment of attraction versus repellency, whereas a plant-vs-plant design would only reveal relative preference between two odor sources. We acknowledge that this design cannot establish whether herbivory caused a host-choice reversal relative to uninfested plants, and our conclusions are therefore limited to density-associated changes in olfactory response relative to clean air. Ten minutes prior to each olfactory assay, all *E. pirisuga* individuals, exuviae, and excreta were gently removed from the infested plants using a soft aspirator, with care taken to avoid additional mechanical damage to leaf tissues. Only plant-derived volatiles were present in the odor source chamber during behavioral testing. To verify that insect-derived cues alone do not confound the observed plant-mediated effects, a pilot bioassay was conducted prior to the formal experiment: odors from *E. pirisuga* nymphs (above 3rd instar, at the maximum density equivalent to 160 individuals) were presented against clean air in the Y-tube olfactometer with no plant material in either arm. No significant preference or avoidance by *E. obliqua* was detected (χ² = 0.42, P = 0.517), confirming that insect body odors, exuviae and frass alone do not drive measurable behavioral responses. A mechanical damage control group was included to distinguish wound-induced volatiles from herbivore-specific HIPVs. Tender tea shoots were punctured with sterilized insect pins to simulate piercing-sucking damage; the total damaged leaf area was calibrated to match the injury caused by 10 late-instar *E. pirisuga* nymphs feeding for 24 h (consistent with the life stage used in infestation treatments). The mechanical damage treatment was tested following the same Y-tube olfactometer protocol.

Ten adult *E. obliqua* individuals were individually introduced into the main tube of the Y-tube. The movement of the insects towards either the HIPV-emitting side or the clean-air side was observed and recorded for 10 minutes. Each independently infested tea plant served as one biological replicate. Five biological replicates (i.e., five independently infested plants) were established for each infestation density, and 10 *E. obliqua* adults were tested per plant as technical subsamples, yielding a total of 50 adults tested per treatment. To eliminate positional bias, the odor source connections of the two Y-tube arms were swapped after every five insects tested.

### 2.4 Olfactometer tests with synthetic chemical of candidate compounds

Candidate behaviorally active volatile compounds were selected based on two quantitative criteria from the GC-MS dataset: (1) their relative contents showed statistically significant differences across *E. pirisuga* infestation densities (P < 0.05); and (2) their relative peak area ratio to the internal standard was greater than 50% in at least one infestation treatment. Accordingly, β-ocimene (confirmed as (E)-β-ocimene), methyl jasmonate (MeJA), and methyl salicylate (MeSA) were selected as candidate behaviorally active volatiles. Synthetic compounds with a nominal purity of ≥ 98% were obtained from Macklin Biochemical Co. Ltd. (Shanghai, China). No additional purity verification was performed in this study.

The olfactory tests with these synthetic compounds were conducted using the same method as described above. Different concentrations of each chemical (0, 1, 10, 100, and 1000 mL/L) were prepared in hexane. A filter paper strip was loaded with 10 μL of the chemical solution and placed in one arm of the Y-tube. A filter paper strip loaded with 10 μL of hexane (solvent) was placed in the control arm, following the same equilibration procedure as the treatment arm. The actual amounts of compound applied at the highest concentration (1000 mL/L, i.e., undiluted compound) were: β-ocimene, 8.0 mg (58.7 μmol; MW = 136.23 g/mol, density ≈ 0.80 g/mL); MeJA, 10.3 mg (45.9 μmol; MW = 224.30 g/mol, density ≈ 1.03 g/mL); and MeSA, 11.8 mg (77.6 μmol; MW = 152.15 g/mol, density ≈ 1.18 g/mL). These laboratory doses define the full dose-response range under controlled conditions and substantially exceed the estimated natural emission rates from individual tea plants; these assays therefore demonstrate laboratory behavioral activity but do not directly establish ecological relevance under field conditions. This test was replicated 10 times.

### 2.5 Statistical analysis

All data obtained from the olfactory tests and GC-MS analysis were subjected to statistical analysis. For the GC-MS volatile profiles, global differences in volatile blend composition across infestation densities were assessed using permutational multivariate analysis of variance (PERMANOVA) based on Bray–Curtis dissimilarities with 9999 permutations, followed by principal component analysis (PCA) for ordination visualization (Hervé et al., 2018). For individual volatile compounds, relative contents across treatments were first tested for normality using the Shapiro–Wilk test and for homogeneity of variances using Levene’s test. Normally distributed data were analyzed using one-way ANOVA followed by Tukey’s HSD *post hoc* test for multiple comparisons. Non-normally distributed data were analyzed using the Kruskal–Wallis test followed by Dunn’s *post hoc* test with Bonferroni correction. For the olfactory test data, the proportion of *E. obliqua* individuals choosing each odor source was analyzed using a generalized linear mixed model (GLMM) with a binomial error distribution and logit link function. Infestation density (or compound concentration) was included as a fixed effect, and the individual odor-source plant was included as a random effect to account for the non-independence of moths tested against the same plant (technical subsamples). The significance of the fixed effect was assessed using a likelihood ratio test. For pairwise comparisons within each treatment, chi-square goodness-of-fit tests against an expected 50:50 choice ratio were performed on the responder data only (non-responding individuals excluded), so that the proportions presented correspond to the data used in the statistical analyses. All statistical analyses were carried out using R (version 4.3.1, vegan and lme4 packages) and SPSS 25.0, and differences were considered significant at a P-value < 0.05.

## 3 Results

### 3.1 Behavioral responses of *Ectropis obliqua* to *Empoasca pirisuga* infested plant

We evaluated the olfactory preference of adult *E. obliqua* (2–3 day-old virgin females) to tea plant volatiles induced by graded densities of *E. pirisuga* using a Y-tube olfactometer, with clean air set as the reference odor source. A generalized linear mixed model (GLMM) with plant as a random effect revealed a significant effect of infestation density on *E. obliqua* choice (likelihood ratio test, χ² = 89.32, P < 0.001). Choice proportions were calculated based on responding individuals only, consistent with the statistical analysis. A mechanical damage (MD) treatment was additionally included to disentangle the effects of general physical wounding from herbivore-specific induced plant volatiles.

As showed in **Figure 1**, uninfested tea plants (0 insects per plant) exhibited significant basal attractiveness to *E. obliqua* adults relative to clean air: among responders, 69.0% selected the plant volatiles and 31.0% chose clean air (χ² = 6.10, P = 0.014). Low-density infestation significantly enhanced attractiveness. At 10 insects per plant, 88.1% of responders chose the HIPV arm and 11.9% chose clean air (χ² = 24.38, P < 0.001), representing peak attractiveness. At 20 insects per plant, 81.1% selected the HIPV side and 18.9% selected clean air (χ² = 14.30, P < 0.001). As density increased further, attraction diminished. At 40 insects per plant, 61.5% chose HIPV and 38.5% chose clean air (χ² = 2.08, P = 0.150), and at 80 insects per plant, 44.1% chose HIPV and 55.9% chose clean air (χ² = 0.47, P = 0.493), neither differing significantly from random choice. At 160 insects per plant, the response shifted to strong repellency: only 2.4% of responders chose HIPV while 97.6% chose clean air (χ² = 37.10, P < 0.001). For the mechanical damage treatment, 64.3% of responders selected MD plant volatiles and 35.7% selected clean air (χ² = 3.43, P = 0.064), which was not significantly different from the uninfested control (GLMM *post hoc*, P = 0.682) but was significantly lower than the 10-insect treatment (GLMM *post hoc*, P < 0.001). This indicates that the enhanced attraction at low herbivore density was driven by herbivore-specific HIPVs rather than general wound-induced volatiles. Overall, *E. obliqua* exhibited a clear density-dependent olfactory response to *E. pirisuga*-induced tea volatiles relative to clean air: low infestation densities enhanced plant attractiveness, whereas high densities reversed the effect to strong repellency. Because clean air rather than uninfested plants was used as the reference, these results demonstrate density-associated changes in the absolute behavioral valence of the induced blends, but do not directly establish a host-choice reversal relative to intact plants.

**Figure 1:**
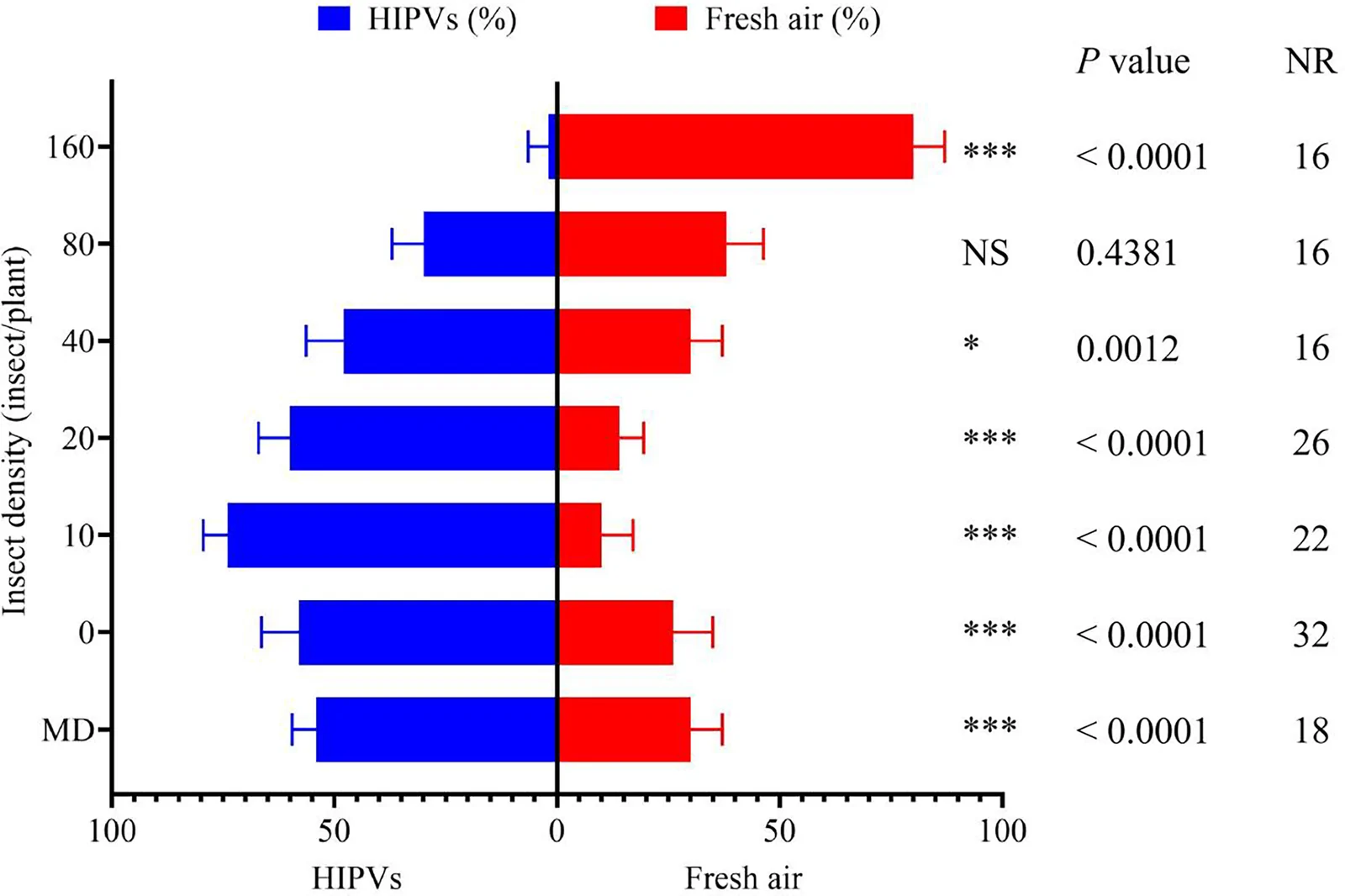
Olfactory preference of *Ectropis obliqua* adults to tea plant volatiles induced by different densities of *Empoasca pirisuga*. The bidirectional bar chart shows the percentage of responding individuals choosing the herbivore-induced plant volatile (HIPV) side (left) and the clean air side (right); non-responding individuals were excluded from percentage calculations. Each density treatment comprised five independently infested plants (biological replicates), with 10 *E. obliqua* adults tested per plant (50 total per treatment); sample sizes for each bar are provided in the Results text. Statistical significance was determined by chi-square goodness-of-fit test against an expected 50:50 choice ratio. ***P < 0.001; *P < 0.05; ns, no significant difference (P ≥ 0.05). A mechanical damage (MD) treatment and an uninfested (0 insects/plant) control were included; GLMM *post hoc* comparisons revealed no significant difference between MD and uninfested plants (P = 0.682), while MD plants were significantly less attractive than 10-insect infested plants (P < 0.001). NR means non-response rate.

### 3.2 Identification of *E. pirisuga* induced tea plant volatiles

The GC-MS analysis identified and quantified the volatile compounds emitted by tea plants infested with different densities of *E. pirisuga* (**Table 1**). PERMANOVA revealed a significant effect of infestation density on overall volatile blend composition (F = 8.73, P = 0.001, 9999 permutations), indicating that herbivore density strongly structures the HIPV profile. PCA ordination confirmed a clear density-dependent clustering trajectory, with the first two principal components explaining 71.7% of total variance (**Figure 2**). Loading analysis identified β-ocimene, MeSA and MeJA as the top three compounds driving treatment separation.

**Table 1:** Volatile components analysis for different number of *Empoasca pirisuga* induced tea plant volatile in 24 h.

Compounds	Relative content to nonyl acetate (IS)					
	Control (0 insect/plant)	10 insect/plant	20 insect/plant	40 insect/plant	80 insect/plant	160 insect/plant
beta.-Myrcene	2.15 ± 0.24bc	2.37 ± 0.17b	2.19 ± 0.25b	3.17 ± 0.09a	1.03 ± 0.05d	1.66 ± 0.18c
D-Limonene	1.76 ± 0.23b	2.81 ± 0.13a	1.34 ± 0.15bc	1.53 ± 0.32b	2.76 ± 0.33a	0.84 ± 0.06c
p-Cymene	3.19 ± 0.30a	1.74 ± 0.09cd	1.68 ± 0.14d	2.30 ± 0.25bc	2.46 ± 0.24b	2.46 ± 0.15b
Benzaldehyde	1.97 ± 0.25abc	1.56 ± 0.07c	1.70 ± 0.17c	2.51 ± 0.49a	1.78 ± 0.24bc	2.46 ± 0.13ab
β-Ocimene	78.87 ± 6.59e	317.09 ± 19.68a	262.09 ± 32.56ab	246.36 ± 14.77bc	194.47 ± 22.50cd	166.04 ± 33.09d
gamma.-Terpinene	ND	1.74 ± 0.30ab	1.35 ± 0.19b	1.92 ± 0.11a	1.67 ± 0.08ab	1.80 ± 0.20ab
1,6-Octadien-3-ol,3,7- dimethyl	ND	2.22 ± 0.17b	1.33 ± 0.10c	3.13 ± 0.36a	1.07 ± 0.04c	2.39 ± 0.01b
Phenylethyl Alcohol	ND	1.73 ± 0.27b	2.26 ± 0.13a	2.28 ± 0.10a	2.28 ± 0.06a	2.49 ± 0.07a
Dodecane	ND	1.30 ± 0.12b	1.24 ± 0.10b	2.13 ± 0.27a	2.19 ± 0.15a	2.16 ± 0.19a
3-Methyl-3-cyclo hexen-1-one	24.99 ± 5.00a	0.81 ± 0.02b	ND	ND	ND	ND
Butanoic acid,3-hexenyl ester,(Z)-	ND	1.80 ± 0.28bc	2.47 ± 0.17a	2.16 ± 0.17ab	1.48 ± 0.16c	2.27 ± 0.13ab
DecaNDl	2.10 ± 0.98a	0.28 ± 0.03b	0.12 ± 0.02b	0.23 ± 0.02b	0.29 ± 0.04b	0.12 ± 0.01b
Indole	ND	2.16 ± 0.21b	0.67 ± 0.10d	3.40 ± 0.21a	1.14 ± 0.06c	3.19 ± 0.08a
Tridecane	ND	2.51 ± 0.12ab	2.72 ± 0.15a	2.12 ± 0.30b	2.81 ± 0.30a	2.46 ± 0.10ab
Acetic acid, octyl ester	ND	1.85 ± 0.41b	2.95 ± 0.47a	2.23 ± 0.10ab	2.60 ± 0.38ab	2.29 ± 0.11ab
MeJA	ND	ND	ND	ND	24.76 ± 4.36b	75.54 ± 3.24a
Caryophyllene	0.21 ± 0.04a	0.11 ± 0.01b	ND	ND	ND	ND
alpha.-Farnesene	16.83 ± 4.78bc	15.18 ± 1.79c	14.44 ± 0.41c	24.60 ± 0.58a	25.65 ± 2.16a	23.18 ± 1.60ab
Hexanoic acid, 3-hexenyl ester	ND	13.49 ± 0.59c	16.08 ± 2.11c	18.82 ± 0.33bc	27.12 ± 3.50a	21.80 ± 2.55ab
MeSA	ND	92.67 ± 5.39a	81.07 ± 1.72a	69.06 ± 8.47b	51.07 ± 2.55c	ND
Hexadecane	ND	0.35 ± 0.04a	0.12d	0.21 ± 0.02c	0.25 ± 0.02bc	0.31 ± 0.03ab
Cedrol	ND	0.31 ± 0.03a	0.27a	0.17 ± 0.02b	0.28 ± 0.03a	0.18 ± 0.01b
(Z)-3-hexene-1- ol	ND	0.24 ± 0.05ab	0.30 ± 0.01a	0.17 ± 0.02b	0.22 ± 0.01b	0.22 ± 0.03b

Data are reported as the mean relative content ± standard deviation (SD) of values of three replicates. Means followed by different letters are significantly different (P < 0.05, one-way ANOVA, Least Significance Difference test), ‘ND’ means undetected. Values are mean relative content (% of internal standard, nonyl acetate) ± SD (n = 3 biological replicates per density). Different lowercase letters within a row indicate significant differences among infestation densities (one-way ANOVA followed by Tukey HSD *post hoc* test, P < 0.05). ND = not detected. The internal standard was 10 μL of nonyl acetate (10 ng/μL in n-hexane) pipetted onto filter paper inside the collection chamber during SPME headspace sampling.

**Figure 2:**
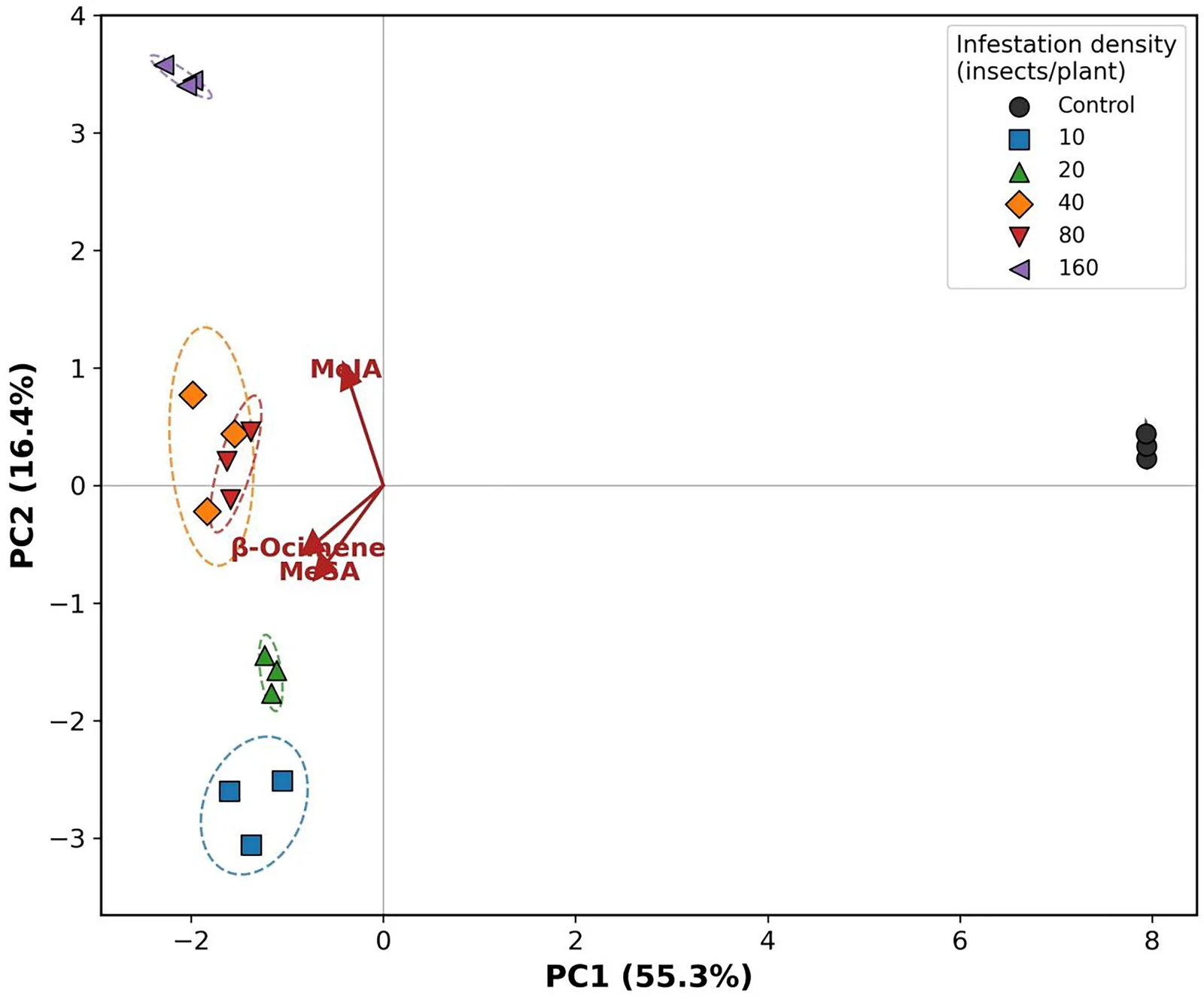
Principal component analysis (PCA) of tea plant volatile blends induced by different densities of *Empoasca pirisuga*. Score plot shows individual biological replicates of each infestation density, with 95% confidence ellipses for each treatment group. Red arrows indicate the loading vectors of the three key candidate compounds (β-ocimene, MeSA, MeJA). Percentages in parentheses represent the proportion of total variance explained by each principal component. PERMANOVA confirmed a significant effect of infestation density on volatile blend composition (F = 8.73, P = 0.001).

At the individual compound level, β-ocimene showed a remarkable density-dependent change. In the control group, its relative content was 78.87 ± 6.59% (mean ± SD), which increased significantly to 317.09 ± 19.68% at 10 insects per plant (P < 0.001) and then gradually decreased with increasing *E. pirisuga* density. Methyl jasmonate (MeJA) was undetected in the control, 10, 20, and 40 insects per plant treatments. It emerged at 80 insects per plant with a relative content of 24.76 ± 4.36% and increased significantly to 75.54 ± 3.24% at 160 insects per plant (P < 0.001). Methyl salicylate (MeSA) was below the limit of detection in the control and 160 insects per plant treatments. It reached its highest relative content of 92.67 ± 5.39% at 10 insects per plant and then decreased with increasing *E. pirisuga* density. Other compounds such as gamma-Terpinene, 1,6-Octadien-3-ol,3,7-dimethyl, and Phenylethyl Alcohol were undetected in the control but present in the infested treatments, with relatively stable or slightly varying relative contents across densities.

Overall, infestation by *E. pirisuga* induced progressive, density-dependent compositional shifts in tea plant volatile blends, with β-ocimene, MeJA and MeSA identified as the major density-responsive candidate compounds. One-way analysis of variance (ANOVA) was performed for all 22 quantified compounds to test the effect of infestation density. Of these, β-ocimene, MeJA, and MeSA showed the most pronounced and statistically significant density-dependent patterns (P < 0.05; **Table 1**; **Figure 2**). The remaining 19 compounds either showed no significant effect of infestation density (P ≥ 0.05) or exhibited variable emission patterns without a consistent density-dependent trend, and were therefore not pursued further as candidate behaviorally active volatiles. The relative abundance patterns of the three key candidate compounds across infestation densities are presented graphically in **Figure 3**.

**Figure 3:**
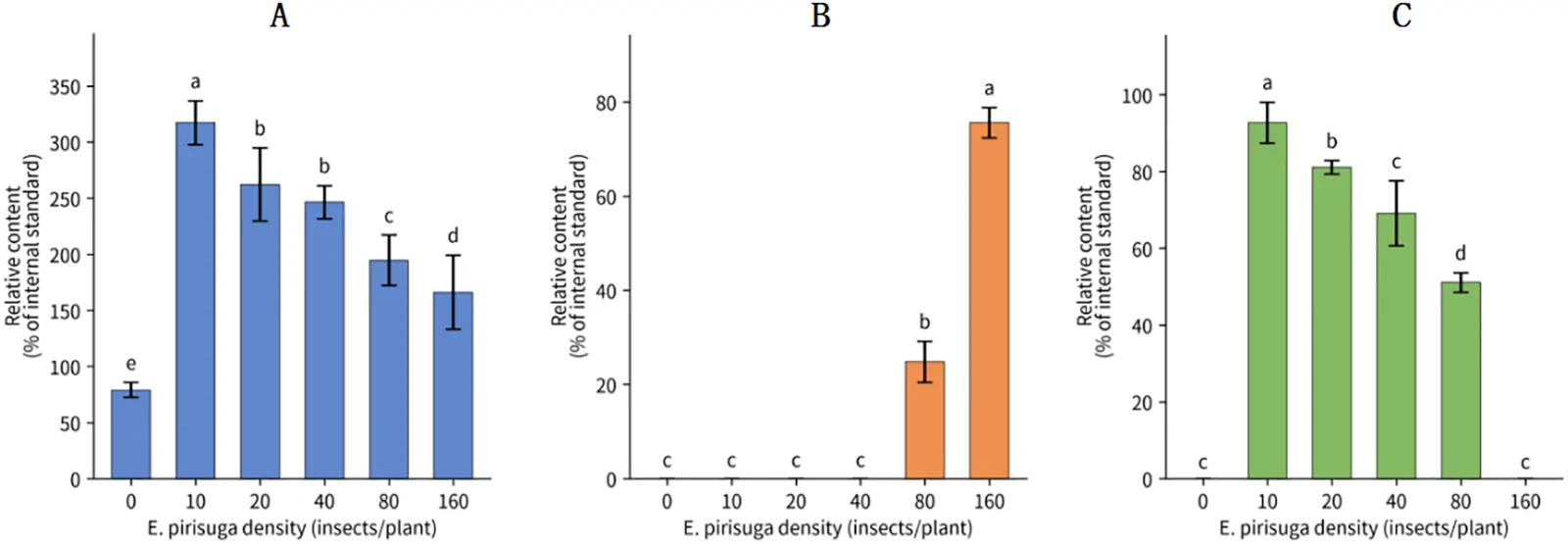
Relative content of the three key candidate compounds across *E. pirisuga* infestation densities. **(A)** β-ocimene; **(B)** methyl jasmonate (MeJA); **(C)** methyl salicylate (MeSA). Bars represent mean relative content (% of internal standard, nonyl acetate) with error bars indicating ± SD (n = 3 biological replicates per density). Different lowercase letters above bars indicate significant differences among infestation densities (one-way ANOVA followed by Tukey HSD *post hoc* test, P < 0.05). ND, not detected.

### 3.3 Behavioral responses of *E. obliqua* to candidate compounds

Based on the GC–MS volatile profiling results, β-ocimene, methyl jasmonate (MeJA), and methyl salicylate (MeSA) were selected as candidate behaviorally active volatiles. Choice patterns and statistical significance are presented in **Figure 4**.

**Figure 4:**
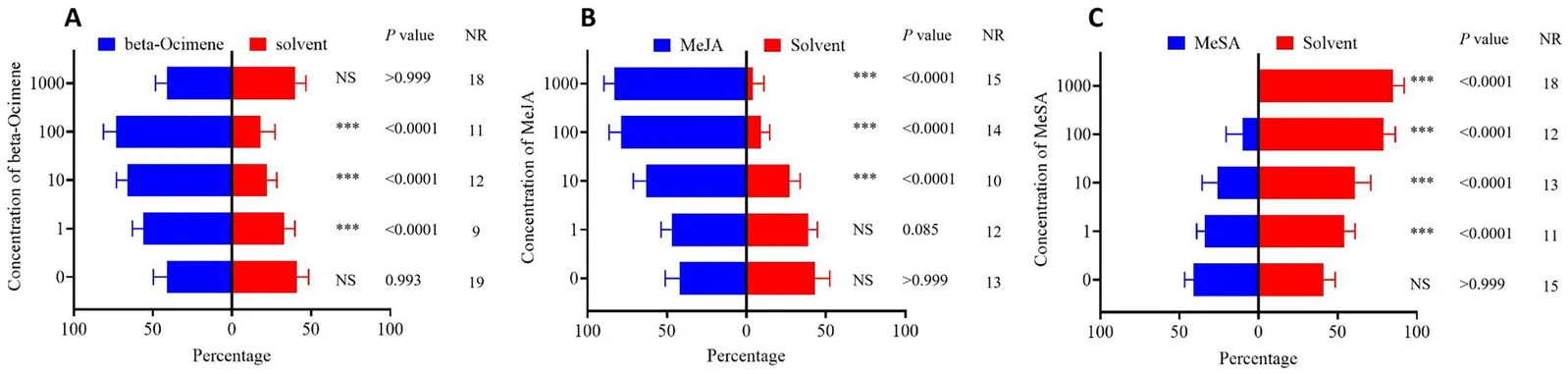
Olfactory preference of *Ectropis obliqua* adults to three candidate synthetic compounds relative to hexane solvent control. **(A)** β-ocimene; **(B)** methyl jasmonate (MeJA); **(C)** methyl salicylate (MeSA). Bidirectional bars show the percentage of responding individuals choosing the compound-treated side (right) and the solvent control side (left) across five concentrations (0, 1, 10, 100, and 1000 mL/L); the Y-axis represents the concentration of each synthetic compound in mL/L. Non-responding individuals were excluded from percentage calculations. Each compound was tested with five biological replicates at each concentration, with 10–11 virgin female *E. obliqua* adults per replicate; total responding individuals per concentration are reported in the Results text (Section 3.3). Statistical significance was determined by chi-square goodness-of-fit test against an expected 50:50 choice ratio. ***P < 0.001; ns, no significant difference (P ≥ 0.05). P-value labels in panels **(B, C)** have been corrected from the previous version; the highest concentrations of MeJA (1000 mL/L, complete attraction) and MeSA (1000 mL/L, complete repellency) are both highly significant (P < 0.001).

For β-ocimene (**Figure 4A**), the 0 mL/L solvent baseline showed equal choice between the two arms among responders (50.0% vs. 50.0%, χ² = 0.00, P = 1.000), verifying that hexane itself did not bias insect orientation. At 1 mL/L, β-ocimene induced a weak attractive trend (63.6% vs. 36.4%, χ² = 3.27, P = 0.070). Significant attraction emerged at 10 mL/L (75.0% vs. 25.0%, χ² = 11.00, P < 0.001) and intensified at 100 mL/L (80.0% vs. 20.0%, χ² = 16.20, P < 0.001). At 1000 mL/L, the attractive effect disappeared (50.0% vs. 50.0%, χ² = 0.00, P = 1.000).

MeJA exhibited a steady, monotonic concentration-dependent attractive effect on virgin female *E. obliqua* (**Figure 4B**). The 0 mL/L solvent control showed no inherent preference (48.8% vs. 51.2%, χ² = 0.02, P = 0.879). At 1 mL/L, MeJA caused a slight but non-significant attractive trend (54.5% vs. 45.5%, χ² = 0.36, P = 0.546). Significant attraction was detected at 10 mL/L (69.6% vs. 30.4%, χ² = 7.04, P = 0.008). Attractiveness intensified at 100 mL/L (90.9% vs. 9.1%, χ² = 29.45, P < 0.001) and reached maximum at 1000 mL/L (95.5% vs. 4.5%, χ² = 36.36, P < 0.001).

In contrast, MeSA exerted a concentration-dependent repellent effect on virgin female *E. obliqua* (**Figure 4C**). The 0 mL/L solvent baseline showed equal choice (50.0% vs. 50.0%, χ² = 0.00, P = 1.000). At 1 mL/L, MeSA induced a weak repellent trend (38.6% vs. 61.4%, χ² = 2.27, P = 0.132). Significant repellency was observed at 10 mL/L (30.2% vs. 69.8%, χ² = 6.72, P = 0.010). Repellency strengthened at 100 mL/L (11.1% vs. 88.9%, χ² = 27.22, P < 0.001) and was complete at 1000 mL/L (0.0% vs. 100.0%, χ² = 42.00, P < 0.001).

Overall, the synthetic compound bioassays confirmed that β-ocimene and MeJA function as individual attractants, whereas MeSA functions as an individual repellent for virgin female *E. obliqua* under laboratory conditions. These three compounds are consistently treated as candidate behaviorally active volatiles throughout this manuscript; their divergent dose–response patterns may contribute to the density-dependent behavioral shift observed in the whole-plant HIPV assays, but synergistic or antagonistic interactions within the natural blend require further validation via mixture and omission experiments.

## 4 Discussion

The findings of this study provide insights into the complex interactions between *Empoasca pirisuga*, *Ectropis obliqua*, and tea plants under controlled laboratory conditions, specifically highlighting the association between HIPV profiles and the olfactory behavior of virgin female *E. obliqua*. Below we discuss the potential mechanisms underlying the observed density-dependent patterns and their ecological implications.

The GC-MS analysis results support the observed behavioral changes and provide a potential mechanistic basis for the density-dependent shift. β-Ocimene showed a significant increase in relative content at 10 insects per plant compared to the control, and then a gradual decrease with increasing *E. pirisuga* density. Given that β-ocimene was attractive to virgin female *E. obliqua* in the synthetic chemical tests, its initial increase at low *E. pirisuga* densities could be associated with *E. obliqua*’s attraction to the HIPVs. MeJA, which was undetected at low *E. pirisuga* densities and appeared at 80 insects per plant, increased significantly at 160 insects per plant. The JA signaling pathway is a well-characterized mediator of plant defense against herbivores across diverse plant systems, and MeJA is a common component of HIPV blends (Amo et al., 2022; Teng et al., 2023). Since MeJA was attractive to virgin female *E. obliqua* in the synthetic chemical tests, its late appearance might play a role in the complex balance of attraction and repulsion, potentially as part of a late-stage defense response that, along with other compounds, contributes to the overall change in *E. obliqua*’s behavior. MeSA, which is commonly found in HIPVs induced by sucking insects (Xiao et al., 2022; Yang et al., 2023; Athanasiadou and Meyhöfer, 2025), reached its highest relative content at 10 insects per plant and then decreased. As MeSA was repellent to virgin female *E. obliqua* in the synthetic chemical tests, its high content at low *E. pirisuga* densities might be counteracted by other attractive compounds, particularly the strongly elevated β-ocimene. At higher densities, the combined effect of decreasing β-ocimene, accumulating MeJA, and the appearance of additional defensive volatile components could lead to the observed repellency. In tea plants, the jasmonic acid (JA) and salicylic acid (SA) signaling pathways are core regulators of herbivore-induced volatile biosynthesis, and CsJMT and CsSAMT are key enzymes controlling MeJA and MeSA emission (Deng et al., 2017; Wan et al., 2025). We emphasize that β-ocimene, MeJA and MeSA are treated as candidate behaviorally active volatiles in this study; their individual behavioral activity does not confirm their causal role in mediating the whole-blend response. Future work should examine the temporal expression dynamics of JA/SA pathway genes, identify olfactory binding proteins and odorant receptors in *E. obliqua*, and validate the functional role of these compounds through reconstructed-blend and omission assays combined with GC-EAD/EAG analysis.

In the olfactory tests with *E. pirisuga*-infested plants, *E. obliqua*’s responses to HIPVs varied with the density of *E. pirisuga*. At 10 insects per plant, the HIPVs were highly attractive to *E. obliqua*. One possible explanation for this attraction is that low-level herbivory may induce a volatile profile that signals a nutritionally suitable or weakly defended host. This phenomenon is not without precedent: Carroll et al. (2006, 2008) demonstrated that *Spodoptera frugiperda* larvae are attracted to volatiles from conspecific-damaged maize and cowpea seedlings, despite the potential for cannibalism and competition, and proposed that herbivore-induced volatiles may serve as reliable indicators of host plant quality or suitability. Similarly, in our system, low-density *E. pirisuga* infestation may induce a blend that *E. obliqua* associates with a host that is sufficiently vigorous to support larval development. From a mechanistic standpoint, phloem-feeding herbivores such as leafhoppers are known to deliver salivary effectors that can suppress or modulate plant defense responses (Gu et al., 2024; Pan et al., 2024). At low infestation densities, these effectors may attenuate the plant’s defensive response, resulting in a volatile blend dominated by attractive compounds such as β-ocimene. As *E. pirisuga* density increased, the attractiveness of the HIPVs to *E. obliqua* decreased, and at 80 and 160 insects per plant, the HIPVs became repellent. This shift may reflect a transition from suppressed to activated plant defense: at high infestation pressure, the cumulative damage overwhelms the effector-mediated suppression, triggering a robust defensive response characterized by the accumulation of MeJA and restructuring of the entire volatile blend (Shiojiri et al., 2010). Additionally, extremely high infestation densities (e.g., 160 nymphs per plant) may directly reduce plant quality by depleting phloem resources and impairing photosynthetic capacity, making these plants poor-quality hosts for *E. obliqua* larvae (Aguirrebengoa et al., 2023). The repellency observed at high densities may therefore reflect *E. obliqua*’s avoidance of compromised host plants, a strategy that would be adaptive under natural conditions. Previous studies have demonstrated density-dependent herbivore response patterns in other insect–host plant systems (Shiojiri et al., 2010; Aguirrebengoa et al., 2023; Cai et al., 2014). The lack of a significant difference between mechanically damaged plants and uninfested controls (GLMM *post hoc*, P = 0.682), combined with the significantly lower attraction of MD plants compared with 10-insect infested plants (P < 0.001), confirms that the density-dependent behavioral shift of *E. obliqua* is associated with herbivore-specific HIPVs rather than physical wounding alone. It is important to note that all behavioral assays used 2–3 day-old virgin female *E. obliqua*. Previous studies have shown that mating status significantly affects olfactory responses in this species; Jing et al. (2021) reported that β-ocimene emitted from neighboring tea plants repelled mated *E. obliqua* females, whereas our study found that β-ocimene attracted virgin females at moderate concentrations. This discrepancy likely reflects mating-status-dependent shifts in olfactory coding and behavioral priorities, and our results should not be directly extrapolated to oviposition behavior, host colonization, or population-level pest dynamics. Additionally, because clean air was used as the reference control rather than uninfested plants, our results demonstrate density-associated changes in the absolute behavioral valence of induced blends relative to a neutral baseline, but do not directly establish that herbivory caused a host-choice reversal relative to intact tea plants.

The apparent contradiction—that repellent MeSA is most abundant at low infestation density when the blend is overall attractive—can be explained by the integrated action of multiple volatile components. At 10 insects per plant, β-ocimene increased approximately 3-fold relative to the control, and its strong attractive effect likely outweighs the repellent effect of MeSA, resulting in net attraction. As infestation density increases, β-ocimene gradually declines, MeJA accumulates sharply at high densities, and additional defensive volatile components are released. The combined repellent effect of the altered blend eventually exceeds the attractive effect, driving the behavioral shift. It should be emphasized that single-compound bioassays cannot fully replicate the effects of natural blends; synergistic and antagonistic interactions among components require further validation via mixture and omission experiments. We also acknowledge that, of the 22 compounds quantified, the majority (19 compounds) did not show consistent density-dependent emission patterns, with some exhibiting variable or fluctuating levels across infestation densities (e.g., D-limonene, p-cymene, indole). This variability may reflect biological noise, the involvement of these compounds in other physiological processes unrelated to density-dependent defense, or insufficient statistical power given the small number of biological replicates (n = 3). Our analysis therefore focused on the three compounds that showed the most robust and statistically significant density-dependent patterns, while recognizing that the remaining compounds may still contribute to the overall blend in ways that our current experimental design cannot fully resolve.

The presence of other compounds like gamma-Terpinene, 1,6-Octadien-3-ol,3,7-dimethyl, and Phenylethyl Alcohol, which were undetected in the control but present in the infested treatments, also likely contributes to the overall blend of HIPVs. From an ecological perspective, our findings provide laboratory evidence consistent with the hypothesis of density-dependent plant-mediated indirect interactions (PMIIs) in the tea agroecosystem. The valence shift of *E. obliqua* olfactory response—from attraction under low-density *E. pirisuga* infestation to repellency under high-density infestation—may reflect an adaptive chemical response of tea plants to gradient herbivory pressure. At the early stage of leafhopper infestation, moderately elevated β-ocimene and MeSA may signal a partially stressed host that remains suitable for foraging and host selection, potentially allowing *E. obliqua* to aggregate and share the resource. As infestation intensity escalates, the dramatic accumulation of MeJA and the restructuring of the entire volatile blend may indicate a strongly defended or nutritionally compromised host, which could in turn deter *E. obliqua* from approaching to avoid excessive interspecific competition and poor offspring performance. These findings suggest that density-dependent HIPV modulation may influence host-plant selection and foraging behavior of *E. obliqua* under laboratory conditions. However, extrapolation to population dynamics or dominance alternation in natural tea plantations would require additional evidence from oviposition preference, larval performance, interspecific competition, and multi-generational population dynamics studies, which were not measured in the present work. Field studies measuring actual colonization rates, oviposition, and population trajectories are needed to test whether these laboratory observations translate to population-level effects in tea plantations.

From a practical perspective, these findings provide a laboratory-based chemical basis for developing behavior-based sustainable pest management strategies in tea plantations, although field validation is required before practical application. For example, if the release of HIPVs can be manipulated in tea plantations, it may be possible to alter the behavior of *E. obliqua*. Releasing synthetic β-ocimene at low concentrations could potentially attract *E. obliqua* to traps for monitoring or mass trapping. Similarly, using MeSA or other repellent compounds could be explored as a push strategy to keep *E. obliqua* away from tea plants. We emphasize that these management applications are proposed based on laboratory behavioral assays with virgin females and require field testing to confirm efficacy under natural conditions, where mated females, complex background odors, and environmental variables will influence behavioral outcomes.

However, several limitations exist in this study. The findings are based on controlled laboratory conditions, and several factors may limit direct extrapolation to field settings. In natural tea plantations, wind speed, temperature fluctuations, complex background odors from surrounding vegetation, and the presence of natural enemy communities can all alter volatile dispersion and insect behavioral responses. Future field validation should include (1) monitoring *E. obliqua* colonization in field plots with graded *E. pirisuga* infestation densities, (2) field plot testing of slow-release volatiles for push-pull pest management, and (3) multi-year population dynamic surveys to verify the association between HIPV profiles and pest dominance shifts. In addition, this study has the following notable constraints: (1) only individual synthetic compounds were tested, leaving potential synergistic or antagonistic interactions within the natural HIPV blend unexamined; (2) experiments focused solely on adult olfactory orientation of virgin females, without evaluating mated females, oviposition preference, larval performance or population-level fitness outcomes; (3) natural enemies were not incorporated into the experimental framework; (4) clean air was used as the reference control rather than uninfested plants, so the results cannot establish whether herbivory caused a host-choice reversal relative to intact plants; (5) the synthetic compound doses substantially exceed estimated natural emission rates, demonstrating laboratory behavioral activity but not direct ecological relevance; and (6) the three compounds are consistently treated as candidate behaviorally active volatiles, and their causal role in mediating the whole-blend response remains to be confirmed.

## Data Availability

The raw data supporting the conclusions of this article will be made available by the authors, without undue reservation.
